# The associations between magnetic resonance imaging findings and low back pain: A 10-year longitudinal analysis

**DOI:** 10.1371/journal.pone.0188057

**Published:** 2017-11-15

**Authors:** Juichi Tonosu, Hiroyuki Oka, Akiro Higashikawa, Hiroshi Okazaki, Sakae Tanaka, Ko Matsudaira

**Affiliations:** 1 Department of Orthopedic Surgery, Kanto Rosai Hospital, Kanagawa, Japan; 2 Department of Medical Research and Management for Musculoskeletal Pain, 22nd Century Medical and Research Center, Faculty of Medicine, The University of Tokyo, Tokyo, Japan; 3 Department of Orthopedic Surgery, Faculty of Medicine, The University of Tokyo, Tokyo, Japan; Rush University Medical Center, UNITED STATES

## Abstract

**Purpose:**

To conduct a 10-year longitudinal analysis of the relationship between magnetic resonance imaging (MRI) findings and low back pain (LBP).

**Materials and methods:**

Ninety-one volunteers with a history of LBP, but without current LBP were recruited between 2005 and 2006. Participants’ baseline demographics and MRI findings were recorded. All volunteers were invited for a follow-up MRI in 2016; of these, 49 volunteers (53.8%) participated in the follow-up. We enquired whether they had LBP history during the 10 years between the baseline and follow-up examinations. Sagittal T1 and T2-weighted MRI were used to assess the intervertebral space from T12/L1 to L5/S1. We evaluated the presence of disc degeneration by Pfirrmann’s grading system, disc bulging, high intensity zone (HIZ), spondylolisthesis, and any type of Modic changes in the follow-up MRIs. We compared the follow-up MRI findings with the baseline findings; the progress of each finding over the 10 years were also compared between the groups with (n = 36) and without (n = 13) LBP.

**Results:**

Average age of the study participants at follow-up was 44.8 years; 25 were female and 24 were male. Average age, sex, body mass index, and smoking habits of those who did and did not participate in the follow-up study, as well as the demographic characteristics of those who did and did not have LBP history during the 10 years, were not significantly different. Compared with the group without LBP history, the group that had LBP history during the 10 years did not have a significantly increased prevalence of disc degeneration, disc bulging, and HIZ in the follow-up and baseline MRIs. Spondylolisthesis and any type of Modic changes were also not associated with LBP history during the 10 years.

**Conclusions:**

Follow-up MRI findings consistent with Pfirrmann grading ≥4, disc bulging, HIZ, spondylolisthesis, and any type of Modic changes were not associated with LBP history during the 10 years between the baseline and follow-up study. The progresses of these findings were also not associated with the LBP history. In addition, baseline MRI findings were not associated with LBP history during the 10 years; therefore, our data suggest that baseline MRI findings cannot predict future LBP.

## Introduction

Low back pain (LBP) is one of the most common causes of health disability, and continues to be the leading cause of disability over the last decade [1]. A Japanese population study reported that the lifetime prevalence of LBP was >80%, as in other industrialized countries [2].

Magnetic resonance imaging (MRI) is able to identify soft tissue such as disc, nerves, and muscles, which are among the possible sources of LBP; however, in some cases, MRI findings may not necessarily identify the source of LBP. Some reports have shown that disc degeneration was associated with LBP [3,4,5], while others have demonstrated no such relationship [6,7]. It has been suggested that symptoms of chronic LBP are often fluctuating, and that LBP is often demonstrated as a condition with patterns of exacerbation and remission [8]. We have reported that disc degeneration, disc bulging, and high-intensity zone (HIZ) were associated with previous history of LBP, and that patients with these findings are prone to develop severe LBP, unless they did not have current severe LBP [9]. However, these reports were related to cross-sectional studies.

There are a few longitudinal studies regarding the relationship between baseline MRI findings and future LBP [10, 11, 12]; however, there is only one longitudinal study about LBP reporting both baseline and follow-up MRIs [13]. The purpose of this study was to examine the longitudinal associations between MRI findings and LBP history during the 10 years between the baseline and follow-up study. The primary aim of this study was to investigate if the follow-up MRI findings and the progress of each finding were associated with a LBP history during the 10 years. The secondary aim was to investigate if the presence of MRI findings at baseline predicted future LBP.

## Materials and methods

### Study participants

As described in detail previously [9], between September 2005 and March 2006, we recruited volunteers who were also Kanto Rosai Hospital personnel to participate in the study. Ninety-one participants with a history of LBP, but without current LBP at that point were included. We excluded participants who had prior back surgery. LBP was defined as pain localized between the costal margin and the inferior gluteal folds, as depicted in a diagram, with or without lower extremity pain in the past 1 month, according to a previous report [9, 14, 15]. The area was shown diagrammatically on the questionnaire, in accordance to a previous study [9, 15]. LBP was defined as a history of medical consultation for LBP. Medical consultation for LBP is one of the standards for evaluating the severity of LBP; it indicated that the LBP was not mild [16]. In 2016, we invited the 91 volunteers to undergo a follow-up MRI. Of these, we invited 41 incumbent personnel three times via our institution’s intranet. We tried sending postal mails to the rest of the 50 retired personnel because we did not know their e-mail addresses; however, new postal addresses of 15 of these 50 were unknown. Eventually, 49 volunteers participated in the follow-up. We enquired whether they had had LBP history during the 10 years between the baseline and follow-up study, according to the aforementioned definition of LBP. However, we did not enquire whether LBP was a single episode or multiple episodes, if they had had LBP history. The participants’ smoking history was also established. We then compared the demographic data of the participants who did and did not participate in the follow-up study, in order to validate that the participants in the follow-up study were representative of all the participants in the baseline study. This study was approved by the medical/ethics review board of Kanto Rosai Hospital. Informed consent was obtained from all individual participants included in the study.

### Image assessment

MRI was performed using a 1.5T Siemens Symphony scanner (Siemens Healthcare, Erlangen, Germany). The imaging protocol included sagittal T1-weighted and T2-weighted fast spin echo (repetition time: 3,500 ms/echo, echo time: 120 ms, field of view: 300 × 320 mm), similar to our baseline study [9]. Sagittal T1- and T2-weighted images were used to assess the intervertebral space from T12/L1 to L5/S1. We had evaluated the intra-observer and inter-observer variability of assessment of the lumbar MRI scans in the previous study as greater than moderate for all evaluated items [9]; therefore, assessment of the follow-up MRI scans was performed by an orthopedist (J. T.), who was blinded to the participants’ backgrounds. We evaluated the degree of disc degeneration, disc bulging, high-intensity zone (HIZ), spondylolisthesis, and Modic changes at each level of the spine. The degree of disc degeneration on MRI was classified into five grades, based on the Pfirrmann’s classification system [17]. We divided the grading into two groups for the purpose of analysis. We regarded those with grades 1–3 as having no or little disc degeneration, and those with grades 4–5 as having some degree of disc degeneration. Disc bulging was defined as displacement of the disc material, usually by more than 50% of the disc circumference and less than 3 mm beyond the edges of the disc space in the axial plane [18]. As we were only able to evaluate the sagittal planes of the MRI scans, we defined disc bulging as posterior disc displacement less than 3 mm and equivalent to the anterior disc displacement in the sagittal plane, although we could not evaluate more than 50% of the circumference. In the midline slice of sagittal planes, the points of the inferior posterior edge of the upper vertebra and superior posterior edge of the lower vertebra were marked, the two points were connected with a line, and the distance between the top of the posterior bulging disc and the line for evaluating posterior bulging was measured. Anterior bulging was evaluated in the same way. We defined HIZ as an area of brightness or high signal intensity located in the posterior annulus on T2-weighted images, based on previous literature [19]. We defined spondylolisthesis as vertebral slips of >5 mm. Those definitions of the four findings were matched as our baseline study [9]. Modic change was divided as three types according to the definition: low intensity in T1-weighted images and high intensity in T2-weighted images was defined as Modic type 1; high intensity in both T1- and T2-weighted images as Modic type 2; and low intensity in both T1- and T2-weighted images as Modic type 3 [20]. However, in the final analysis, we only evaluated whether any type of Modic changes existed or not.

When a participant had at least one positive finding in any disc level for the item, we regarded the findings of the participant as positive as a whole. Finally, we focused on the relationship between LBP history during the 10 years and the MRI findings at follow-up, baseline, and the progress over 10 years. The progress of each finding was defined as a positive finding at follow-up MRI with negative finding at baseline MRI.

### Statistical analysis

Between-group differences in baseline characteristics were evaluated using the Fisher’s exact test for categorical variables and the Student’s t-test for continuous variables. We compared the differences in MRI findings over 10 years between groups with and without LBP history over 10 years by using Fisher’s exact test. Furthermore, we determined the odds ratios of each item using univariate analyses. Statistical analyses were performed using the JMP 11.0 software program (SAS Institute, Cary, NC, USA). A p value of <0.05 was considered to be significant.

## Results

Of the 91 participants in the baseline study, 41 participants were incumbent and 50 had retired. Of the 41 incumbent participants, 31 participated in the follow-up study, while of the 50 retired participants, 18 participated in the follow-up study. Addresses of 15 retired participants were unknown; thus, we were unable to send postal mails inquiring about their participation. Eventually, of the 91 participants in the baseline study, 49 (54%) participated in the follow-up study. The reasons for no participation are shown (Table 1).

**Table 1:** Details of the participants of the follow-up study.

	Total: 91	Follow-up (+): 49	Follow-up (-): 42	Reason of no participation
Incumbent	41	31	10	Not intending: 5
				No reply: 5
Retired	50	18	32	Not intending: 8
				No reply: 9
				New address unknown: 15

The average ages of those who did and did not participate at the follow-up study were 44.9 and 44.6 years, respectively, which was not significantly different.

There were also no significant differences in sex, bone mass index (BMI), and smoking habit at baseline between the groups (Table 2).

**Table 2:** Demographic data of the participants who did and did not participate in the follow-up study.

		Total: 91	Follow-up (+): 49	Follow-up (-): 42	p-value
Age		44.8 ± 10.7	44.9 ± 9.3	44.6 ± 12.3	0.8966
Sex	Female	48	25 (51.0)	23 (54.8)	0.8337
	Male	43	24 (49.0)	19 (45.2)	
BMI	(kg/m^2^)	21.5 ± 3.8	21.8 ± 4.4	21.1 ± 2.7	0.4051
Smoking habit at baseline	(+)	32 (35.2)	19 (38.8)	13 (31.0)	0.5116

Data are shown as mean ± standard deviation or number of participants (%).

Of the 49 participants in the follow-up study, 36 had a history of LBP during the 10 years between the baseline and follow-up study. Participants’ average age was 44.9 ± 9.3 years; 25 were female and 24 were male; and their average body mass index was 21.8 ± 4.4 kg/m^2^. The average ages of those who did and did not have LBP history over the 10 years were 46.4 and 44.4 years, respectively, which was not significantly different. There were also no significant differences in sex, BMI, and smoking history between the groups (Table 3).

**Table 3:** Demographic data of participants who did and did not have low back pain history during the 10 years between the baseline and follow-up study data are shown as mean ± standard deviation or number of participants (%). LBP; low back pain.

		Total: 49	LBP history: 36	No LBP history: 13	p-value
Age		44.9 ± 9.3	46.4 ± 11.0	44.4 ± 8.7	0.4968
Sex	Female	25	18 (72.0)	7 (28.0)	1.0000
	Male	24	18 (75.0)	6 (25.0)	
BMI	(kg/m^2^)	21.8±4.4	21.7±5.0	22.1±2.6	0.8019
Smoking history	(+)	17 (34.7)	13 (36.1)	4 (30.8)	1.0000

Compared with the group without LBP history during the 10 years, the group that did develop LBP did not have a significantly increased incidence of disc degeneration in at least one spinal level in the follow-up MRIs, compared with the baseline MRIs. There were also no significant differences between the two groups with regards to the progress of disc degeneration over 10 years (Table 4). Additionally, no significant differences in disc bulging in the follow-up and baseline MRI were found between the two groups. Progress of disc bulging was also not significantly related to LBP history during the 10 years (Table 4). There were also no significant differences between the two groups in terms of HIZ in the follow-up and baseline MRI. Progress of HIZ was also not significantly related to LBP history during the 10 years (Table 4). Only two participants exhibited spondylolisthesis in both the follow-up and baseline MRI. There were no significant differences between the two groups in terms of spondylolisthesis in the follow-up and baseline MRI. Of the two participants with spondylolisthesis, one had LBP history during the 10 years, while the other did not. There was no case of progress of spondylolisthesis. Modic type 1 change was identified in only one participant in the follow-up MRI; six participants were found to have type 2, while none had type 3. There were no significant differences between the two groups with regards to Modic changes in the follow-up MRI. Univariate analysis revealed the odds ratios and 95% confidential intervals of each item; however, there were no significant differences in all items (Table 5).

**Table 4:** Magnetic resonance imaging findings at the follow-up and baseline of patients with and without low back pain history during the 10 years between the baseline and follow-up study.

		Total: 49	LBP history: 36	No LBP history: 13	p-value
***Disc degeneration***					
Follow-up MRI	(+)	42 (85.7)	32 (88.9)	10 (76.9)	0.3629
Baseline MRI	(+)	25 (51.0)	19 (52.8)	6 (46.2)	0.7536
Progress	(+)	17 (34.7)	13 (36.1)	4 (30.8)	1.0000
***Disc bulging***					
Follow-up MRI	(+)	37 (75.5)	27 (75.0)	10 (76.9)	1.0000
Baseline MRI	(+)	30 (61.2)	21 (58.3)	9 (69.2)	0.7408
Progress	(+)	10 (20.4)	8 (22.2)	2 (15.4)	0.7095
***High-intensity zone***					
Follow-up MRI	(+)	22 (44.9)	15 (41.7)	7 (53.9)	0.5250
Baseline MRI	(+)	14 (28.6)	11 (30.1)	3 (23.1)	0.7308
Progress	(+)	9 (18.4)	5 (13.9)	4 (30.8)	0.2204

Data are shown as number of participants (%). Pfirrmann grade ≥4 is regarded as disc degeneration. LBP; low back pain, MRI; magnetic resonance imaging.

**Table 5:** Associations between the follow-up magnetic resonance imaging findings and low back pain history during the 10 years according to univariate analyses.

	Odds ratio	95% confidential interval	p-value
Disc degeneration	2.4	0.42–12.78	0.3101
Disc bulging	0.9	0.17–3.77	0.8896
High-intensity zone	0.6	0.17–2.20	0.4500
Spondylolisthesis	0.3	0.01–9.11	0.4700
Modic changes (any)	0.9	0.16–6.81	0.8956

## Discussion

The follow-up study was performed 10 years after the baseline study, with a follow-up rate of 53.8%. Over half of the 91 participants of the baseline study had retired. The follow-up rate of the incumbents was high at 75.6%, while that of the retired group was low at 36.0%. Those who did not intend to participate in the follow-up study might not have adjusted their schedule because only two days could be spared for the follow-up MRI examination. In the institute of the personnel, those who retire leave their new postal address for the office. However, since 10 years had passed, the postal address could have changed once again. Therefore, we could not contact 15 retired participants. Although the follow-up rate was relatively low, the backgrounds of those who did and did not participate in the follow-up study were not significantly different; therefore, we regarded the results of the followed-up participants as representative of the baseline participants.

Both in the baseline and follow-up study, we precisely defined the region of LBP similar to that in our previous study [9], which seemed to be important for standardizing the study protocol for LBP [14,15]. Of the followed-up participants, 73.5% had a history of LBP between the baseline and follow-up study. This was relatively similar to the lifetime prevalence of LBP of approximately 83%, which was based on a population-based survey [2]. Therefore, it can be regarded that the normal population may also have LBP history over 10 years, as in the study participants. There were no significant differences in age, sex, BMI, and smoking history between the groups with or without LBP history during the 10 years. Several previous studies [21, 22] have indicated that smoking was associated with LBP; however, our results were not consistent with their findings.

Pfirrmann grading indicates the degree of disc degeneration [17]. Considering that disc degeneration progresses with advancing age [4], disc degeneration was more frequent in the follow-up MRI assessment compared to the baseline MRI assessment (85.7% vs. 51.0%). Seventeen participants who did not have disc degeneration in the baseline MRI demonstrated disc degeneration in the follow-up MRI. In fact, 76.9% of those who have had no LBP history during the 10 years showed disc degeneration. There have been many reports on the relationship between current LBP and disc degeneration [3, 4, 5], although the results have been controversial. Videman et al showed that disc height narrowing was associated with previous LBP [23], and our previous study showed that disc degeneration was associated with previous LBP [9]. Meanwhile, a systematic review showed that there were not consistent associations between MRI findings and future episodes of LBP [24]. If LBP history during the 10 years was regarded as having previous LBP, our current findings were not consistent with our previous study’s findings, but with the systematic review.

Disc bulging was also more frequent in the follow-up MRI assessment, at 75.5% of all participants, compared to 61.2% in the baseline MRI assessment. Ten of those who did not have disc bulging in the baseline MRI showed disc bulging in the follow-up MRI. While some studies have shown that disc bulging was frequently observed in asymptomatic subjects, and concluded that there was no relationship between disc bulging and current LBP [25, 26], another meta-analysis study demonstrated that there is a strong relationship [5]. As for previous LBP, our previous study demonstrated a significant association between disc bulging and previous LBP [9], while Videman et al had reported no association [23]. The current results showed that there were no relationships about LBP history during the 10 years in the prevalence of the follow-up MRI, the baseline MRI, and the progress of disc bulging, as reported previously.

There were no relationships of the LBP history among the prevalence of the follow up MRI, the baseline MRI and the progress of HIZ, although the frequency of HIZ increased with aging. Aprill and Bogduk reported a strong association between the annular high signal intensity zone and positive provocative discography finding [19], while Schellhas et al found that HIZ was associated with current LBP [27]. Dongfeng et al reported that HIZ may be a specific signal for the inflammatory reaction of a painful disc by their histological study [28]. Conversely, other studies have shown that HIZ was frequently observed in asymptomatic subjects [5, 25, 26]. A longitudinal MRI study showed that 26.6% of HIZ findings resolved and HIZ improved in 14% cases, with no statistical association between HIZ changes and changes in a patient's symptoms [29]. Our results were consistent with the reports that no association was observed.

Spondylolisthesis was considered to be one of the findings of lumbar spine instability [30]; in addition, it was assumed that those who had spondylolisthesis were inclined to have LBP [31]. However, several reports found no significant relationship between spondylolisthesis and current LBP [5, 32]. In the present study, only 2 participants were found to have spondylolisthesis during the baseline MRI assessment; the same 2 participants demonstrated spondylolisthesis during the follow-up assessment, although no progressions were noted. This suggested that no significant relationship was found between spondylolisthesis and LBP history during the 10 years in our study. However, this may be attributed to the small number of spondylolisthesis cases in our sample of participants.

Several reports have found that Modic type 1 change can indicate inflammation of endplates and be related to LBP [3, 33]. As Modic type 1 change was identified in only one case in the follow-up study, we analyzed the relationship between any Modic changes and LBP history during the 10 years. Our results showed that no significant relationship was found, which was inconsistent with previous reports [34, 35].

Brinjikji W et al. reported in their systematic review that disc degeneration, disc bulging, and Modic 1 changes were more prevalent in adults aged 50 years or younger with back pain compared with asymptomatic individuals, because the prevalence in the asymptomatic younger population was much lower [5]. Furthermore, they also demonstrated that disc degeneration, disc bulging, and annular fissure were present in high proportions of asymptomatic individuals, and that this increased with age [36]. Although the average age during the follow-up MRI in our study was 44.8 years, which could be regarded as young, our results were consistent with the systematic review results of an aged population.

There were several limitations to the current study. First, the findings of this study were limited and could not be generalized because of the small sample size. In addition, the follow-up rate was relatively low; however, we were able to demonstrate that the backgrounds of the participants who did and did not participate in the follow-up study were not significantly different. The statistical power was insufficient, however, as it exceeds 0.6 in all disc degeneration types, disc bulging, and high-intensity zones. The power of disc bulging was 0.76, which was the largest among the three. Second, we did not evaluate the Modic changes in the baseline MRI as only sagittal T2-weighted images were analyzed at that stage; therefore, although we evaluated both T1- and T2-weighted images in the follow-up MRI, we were unable to comment on any Modic changes in the baseline MRI. Third, disc bulging and HIZ can sometimes be visible from the posterolateral sides; however, as we only analyzed sagittal images, these findings may have been underestimated. In other words, there is a possibility that the pathology was missed in the zone between the planes of the posterior and anterior vertebral body cortices because only sagittal images were used. Although this limitation had been written in our previous study [9], we also did analyze only sagittal images in the follow-up study, because we preferred same definition of those findings as same as the previous study. Fourth, there was selection bias among our study participants, as they were volunteers from all types of employment at the hospital and did not represent the general population. This was also the limitation in our previous study [9]. Lastly, the lack of specific information about frequency and severity of LBP episodes in the study cohort may be seen as a limitation of this study as well.

## Conclusions

The follow-up MRI findings consistent with Pfirrmann grading ≥4, disc bulging, HIZ, spondylolisthesis, and any type of Modic changes were not associated with LBP history during the 10 years between the baseline and follow-up study. The progress of these findings was also not associated with the LBP history. In addition, baseline MRI findings were not associated with LBP history during the 10 years; therefore, our data suggest that baseline MRI findings cannot predict future LBP.

## Supporting information

S1 File Supporting information. Dataset set of this study.
(XLSX)
